# Comparative Genomics Identifies a Potential Marker of Human-Virulent *Anaplasma phagocytophilum*

**DOI:** 10.3390/pathogens3010025

**Published:** 2014-01-09

**Authors:** Basima Al-Khedery, Anthony F. Barbet

**Affiliations:** 1 Department of Infectious Diseases and Pathology, University of Florida, Gainesville, FL 32611, USA; E-Mail: balkhedery@ufl.edu; 2 Emerging Pathogens Institute, University of Florida, Gainesville, FL 32611, USA

**Keywords:** human anaplasmosis, *Anaplasma phagocytophilum*, comparative genomics, diagnosis, PCR

## Abstract

We have previously described a comparative genome analysis of nine strains of *Anaplasma phagocytophilum* that showed similarity between strains infecting humans and U.S. dogs and a more distant relationship with horse and ruminant strains. This suggested that it may be possible to distinguish human-infective strains using simple DNA sequence-based diagnostic tests. This would be of epidemiologic significance in identifying and tracking the presence of virulent strains in tick vector populations. Further analysis identified a gene that was present in several strains, including U.S. *Ap*-variant 1 (ruminant), MRK (horse), and European sheep, but was deleted in strains infecting U.S. humans and dogs, suggesting that it could be a useful marker of human virulence. A simple PCR test was developed to identify the presence/absence of this gene. The PCR test discriminated *A. phagocytophilum* strains from clinically affected humans and U.S. dogs from the strains more distantly related in genome sequence. This warrants further testing of globally diverse *A. phagocytophilum* strains to examine world-wide conservation of this gene.

## 1. Introduction

*Anaplasma phagocytophilum*, a member of the order Rickettsiales, causes human fatalities in the U.S., Europe and Asia, and also infects multiple animal species. In the U.S., case reports increased from 348 in 2000 to 1,761 in 2010 [1], and the reported hospitalization rate is 36% [2]. Human anaplasmosis (HA) can be treated with antibiotics, but the symptoms, such as headache, fever, and muscle aches are non-specific and can be confused with other common diseases such as the flu, often leading to inappropriate therapy. Increasingly, there are reports of infections transmitted by blood transfusions in the U.S. and Europe [3,4]. The animal species that have been found infected include cattle, sheep, goats, horses, dogs, foxes, cats and rodents. Also, the tick vectors, such as *Ixodes scapularis* and *Ixodes pacificus* in the U.S., are known for the broad range of hosts on which they feed [5]. Different strains of *A. phagocytophilum* have different animal host predilections and not all strains infect all hosts [6]. This complex ecology has made it difficult to assess the risk of transmission to humans and institute control measures.

There is extensive genomic diversity within the *A. phagocytophilum* species. Numerous attempts have been made to link particular genotypes to host-tropism phenotypes with some, although limited, success. For example, in the U.S. a two-base difference in 16S ribosomal RNA has identified some strains infective to either ruminants (known as *Ap*-variant 1) or to mice (and it is thought to humans, which are known as *Ap*-ha strains) [7,8,9]. This method has insufficient discriminatory power, however. Further study of multiple strains worldwide has identified at least fifteen 16S variants, of which *Ap*-ha is one of the most common [10]. However, other variants have also been found in human infections and *Ap*-ha is not limited to humans. Moreover, there are many phenotypically untyped 16S variants in both the U.S. and Europe and multiple variants may co-exist in a single infection [11,12]. Similarly, methods based on polymorphisms in single genes such as *ankA* and *groEL* produce different strain clustering to one another and have failed to definitively categorize human-infective strains.

We recently completed a study analyzing high-throughput gene sequences of *A. phagocytophilum* strains from the U.S. and Europe [13]. The rodent, dog and human strains were similar to one another and to the previously sequenced human-infective strain HZ (98.79%–100% average genome nucleotide identity). The *Ap*-variant 1 strains were different in numerous regions (96.21%–96.28% average nucleotide identity to HZ). Here, we used comparative genomics to identify a gene deletion that has occurred in strains infecting humans and dogs in the U.S. but not in the more distantly related ruminant and horse strains. A simple PCR test was developed to identify the presence/absence of this gene. This marker may aid investigations of the spread by ticks of *A. phagocytophilum* strains which cause disease in humans.

## 2. Results and Discussion

**Figure 1:**
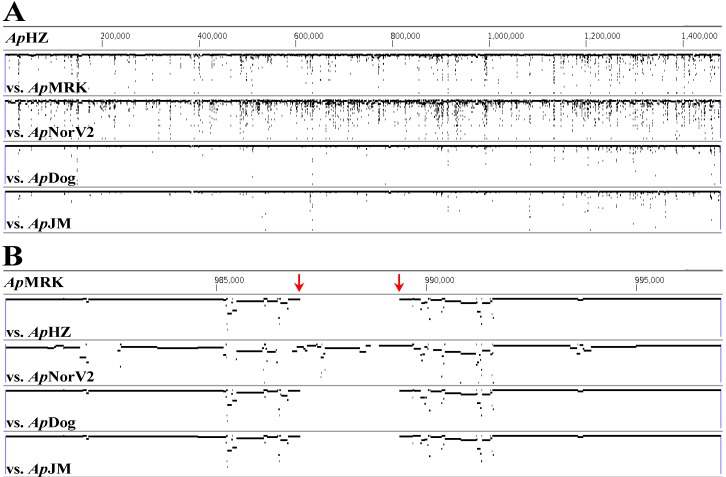
Genome alignments comparing five strains of *A. phagocytophilum*. (**A**) The *Ap*MRK and *Ap*NorV2 strains have numerous regions of lower identity compared to *Ap*HZ than do the *Ap*Dog and *Ap*JM strains. (**B**) There is also a significant genome deletion in *Ap*HZ, *Ap*Dog and *Ap*JM compared to *Ap*MRK and *Ap*NorV2 strains. For each comparison row, the percent identities range from 50 to 100 from the bottom to the top of the rows. Panel B is a zoomed in region of panel A to show the deleted region. The top line in each panel indicates the reference genome, either *Ap*HZ or *Ap*MRK.

**Figure 2:**
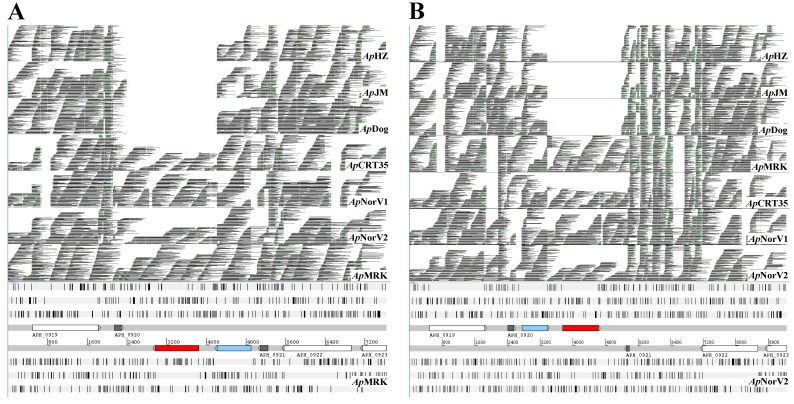
The deleted region encompasses the *drhm* gene (red). The extra copy of APH_0986 is shown in blue. Alignment of Roche/454 raw reads from seven *A. phagocytophilum* strains with the genome segment containing APH_0919 to APH_0923. (**A**) Alignment of reads with *Ap*MRK; (**B**) Alignment of reads with *Ap*NorV2.

**Figure 3:**
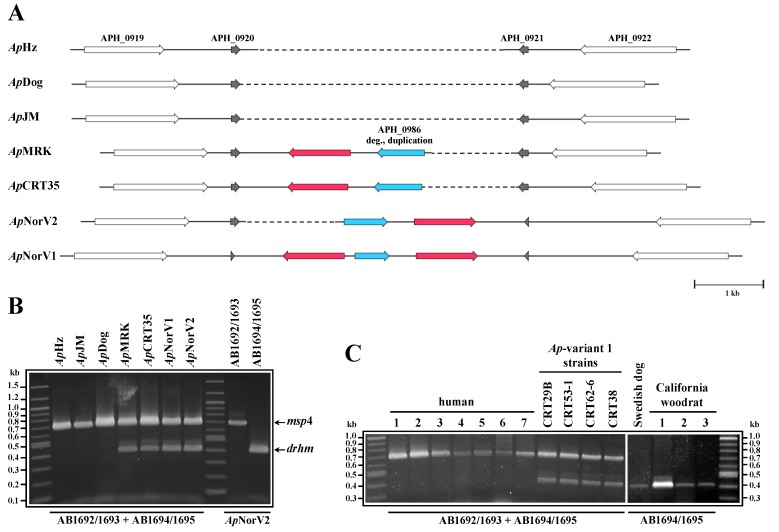
The newly identified *drhm* gene could serve as a potential marker of human-virulent *A. phagocytophilum* strains. (**A**) Comparative maps from 7 *A. phagocytophilum* strains depicting a genomic region spanning two pairs of inversely duplicated ORFs, originally annotated as APH_0919/APH_0920 and APH_0921/APH_0922 in the human *Ap*HZ strain reference genome (CP000235). Sequences corresponding to a degenerate duplication of the ABC transporter gene (APH_0986 in CP000235; blue arrows) and one to two copies of the herein identified *drhm* gene (red arrows) are also present in this region, but only in strains more distantly related in genome sequence to the human *Ap*HZ strain. Maps are drawn to scale. For clarity, sequences related to the *Ap*HZ APH_0920 and APH_0921 ORFs are aligned, and stippled lines are included to indicate gaps relative to the locus in the *Ap*NorV1 strain. (**B** and **C**) A PCR test developed to identify the presence/absence of the *drhm* gene clearly demonstrates the absence of this gene in human strain-related isolates. The human samples used in (**C**) are: 1, *Ap*Webster; 2, *Ap*NY18; 3, 4 and 5, New York patient clinical samples; 6, *Ap*MN1; 7, *Ap*MN2. All template DNAs were obtained from *in vitro* cultures except for *Ap*NorV1, *Ap*NorV2, New York patient clinical samples, Swedish dog and California woodrat samples.

Comparison of genomes by alignment (Figure 1A) showed lower nucleotide identities in *Ap*MRK (horse) and *Ap*NorV2 (sheep) compared to *Ap*HZ, *Ap*JM and *Ap*Dog, agreeing with our previous data [13]. The differences were found throughout the genomes, but were localized particularly to *msp2*/*p44* pseudogenes close to the origin of replication (base #1 in a linear representation of the circular genome [13]). Additionally, we identified genome segments present only in some strains. Figure 1B shows a region that was present in *Ap*MRK and *Ap*NorV2 but was apparently deleted in the other three strains (demarcated with red arrows). This region is sandwiched between two pairs of inversely duplicated open reading frames (ORFs) annotated as APH_0919/APH_0920 and APH_0921/APH_0922 (encoding hypothetical proteins) in the CP000235 reference *Ap*HZ genome (Figure 2 and Figure 3A). We initially identified two novel, relatively large, ORFs in this genome segment, one encoding a degenerate copy of the ABC transporter gene found elsewhere in all the genomes (annotated as APH_0986 in the CP000235 reference *Ap*HZ genome) and a second encoding a gene with no known orthologs in other *A. phagocytophilum* strains, or in the most recent GenBank database. To verify that this was a gene deletion and not caused by misassembly, we compared Roche/454 reads from seven strains directly with this assembled region of *Ap*MRK and *Ap*NorV2 (Figure 2). Clearly, there were reads encompassing this previously unidentified gene in both the *A. phagocytophilum* strains infecting Norwegian sheep, the U.S. ruminant *Ap*CRT35, and the Californian horse (*Ap*MRK) strain. There were no alignable reads from *Ap*HZ (human), *Ap*JM (rodent), or ApDog, indicating that this was indeed a gene deletion in those strains most closely related to the human-infective *Ap*HZ strain.

We developed a PCR assay to investigate the presence or absence of this gene, termed *drhm* (for distantly related to human marker), in other *A. phagocytophilum* strains. The *drhm* gene was present in all U.S. strains tested that were previously identified as the ruminant-tropic *Ap*-variant 1, as well as present in U.S. Californian woodrat strains tested. It was absent in all cultured strains originally derived from human infections in Minnesota or Wisconsin and from blood taken from clinically infected humans in New York state (Figure 3B and 3C). The conserved *msp4* gene was used as an internal positive control to verify the presence of amplifiable *A. phagocytophilum* DNA in all samples.

**Figure 4:**
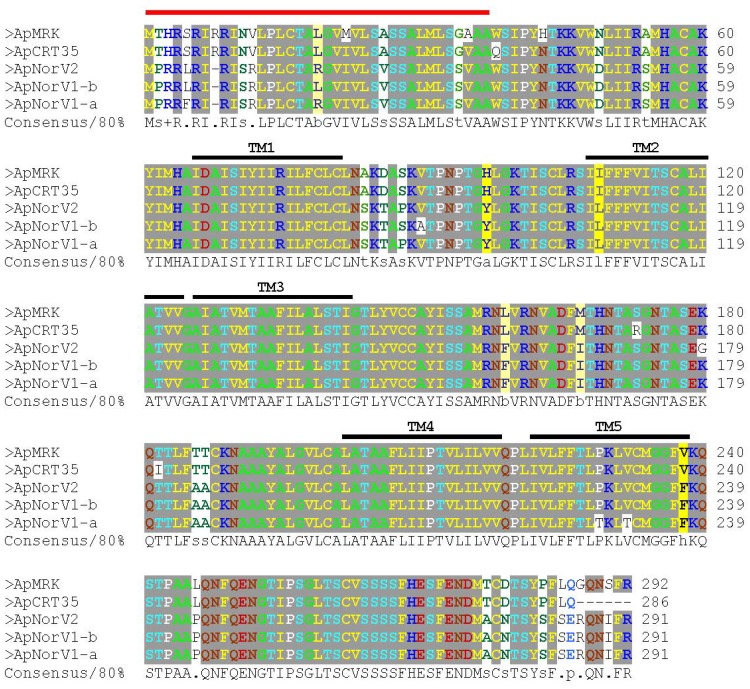
Multiple sequence alignment of amino acid sequences encoded by *drhm* from different strains of *A. phagocytophilum*. The positions of a putative signal peptide (red line) and five predicted TM segments (black lines) are shown.

This genome region appears to be prone to rearrangement, although not as frequent as in *msp2/p44* pseudogenes, as the *drhm* gene is maintained in *A. phagocytophilum* strains derived from Europe as well as from the U.S. Midwest and California. This is most evident from the finding that the deleted segment can occur in either orientation relative to its placement in *Ap*MRK, as well as the existence of two opposing *drhm* genes flanking the degenerate ABC transporter gene copy in the *Ap*NorV1 genome (Figure 3A). It should be noted that no sequences related to the *drhm* gene could be detected in the vicinity of the full-length ABC transporter gene in these strains, nor at any other loci in the assembled *Ap*MRK and *Ap*NorV2 contigs. The putative polypeptides encoded by *drhm* range in identity from 87 to 99% (94 to 99% identity at the nucleic acid level; data not shown) and were strongly predicted by different algorithms to be integral membrane proteins with 5-6 transmembrane (TM) segments (Figure 4).

Further analysis of the amino acid sequence by PSORT suggested a location as inner membrane proteins; some algorithms (e.g., SIGNALP V.2.0) predicted an N-terminal signal peptide from 1–37, but others (e.g., SIGNALP V.4.1) did not. Scanning for motifs at the PRINTS-S Protein Fingerprint Database and other protein profile databases did not yield any significant hits suggestive of a potential function for the DRHM polypeptides.

The high worldwide prevalence of *A. phagocytophilum*, the continuing spread of its vector *Ixodes* ticks into new geographic areas [14], the potential for severe human disease and contamination of blood supplies make this emerging infection a cause for concern. We have shown data suggesting that, at least in the U.S., the presence of the *drhm* gene is indicative of strains that are not infecting humans but are more closely related to the long-known ruminant-infective strains. However, currently the number of strains analyzed at the whole genome level is small and should be expanded to verify this interpretation. One must also consider alternative possibilities. For example, that the gene deletion represents an artifact from adaptation of strains to *in vitro* culture conditions. We believe this to be less likely as the deletion was present in *A. phagocytophilum* DNA derived directly from clinical samples. It is possible that milder human infections may be caused by other strains but have not yet been recognized. Interestingly, in the U.S. the strains identified that cause disease in humans and dogs are similar at the whole genome level. Some data suggest this may not be true globally; *A. phagocytophilum* strains infecting Swedish dogs have not grouped phylogenetically with U.S. dog strains [15]. Similarly, our preliminary data shows that a strain from an infected Swedish dog possesses the *drhm* gene (Figure 3C), unlike the U.S. dog strains analyzed (Figure 3B, and data not shown). There is a significantly lower number of human infections described in Europe compared to the U.S. for reasons that are not totally clear and are likely due to various factors such as levels of tick exposure, infection prevalence and disease surveillance. It is also possible that those strains that are most virulent for humans are not widely distributed in the tick vector and result from recent evolutionary divergence. This could produce localized foci of human infections, similar to those observed. Comparative genomics and the *drhm* PCR test will provide an initial rapid classification of *A. phagocytophilum* strains derived from animal reservoirs and ticks that can be investigated further for human virulence.

## 3. Experimental Section

### 3.1. A. phagocytophilum Strain Origins and Genomic DNA Isolation

The origins of the *A. phagocytophilum* strains used in this study are as follows: *Ap*HZ, human, Westchester, New York, USA [16]; *Ap*NY18, human, New York, USA [17]; *Ap*Webster, human, Wisconsin [18]; *Ap*MN1 and *Ap*MN2, human, Minnesota; *Ap*Dog, dog, Minnesota; *Ap*JM, meadow jumping mouse (*Zapus hudsonius*), Camp Ripley, Minnesota; *Ap*MRK, horse, California [19,20]; *Ap*-CRT35, -CRT29B, -CRT38, -CRT53-1 and -CRT62-6 (*Ap*-variant 1 strains, Camp Ripley tick (*I. scapularis*), Minnesota [21]; *Ap*NorV1 and *Ap*NorV2, sheep, Norway [13,22]. *Ap*Dog, *Ap*CRT35, *Ap*NorV1 and *Ap*NorV2 were previously defined as *Ap*Dog2, *Ap*Var-1, *Ap*NorLamb-V1 and *Ap*NorLamb-V2, respectively [22].

Strains from *in vitro* culture included: *Ap*HZ, *Ap*NY18, *Ap*MN1, *Ap*MN2, *Ap*Webster, *Ap*JM and *Ap*MRK, propagated in HL-60 cells, and *Ap*Dog and *Ap*CRT35, maintained in the *I. scapularis* ISE6 tick cell line. Starter cultures of *Ap*HZ, *Ap*JM, and *Ap*MRK were generously provided by Dr. Ulrike G. Munderloh, as was genomic DNA (gDNA) from *Ap*Dog, *Ap*CRT35, *Ap*MN1 and *Ap*MN2. The three New York patient samples were generously provided by Dr. Susan Wong, NY State Dept. of Health [23], *Ap*NorV1 and *Ap*NorV2 by Drs. Snorre Stuen and Erik G. Granquist, and the *Ap*-variant 1 strains by Dr. Robert F. Massung. *A. phagocytophilum* gDNA was prepared as described previously [22]. Genomic DNA from California dusky-footed woodrats (*Neotoma fuscipes*) was kindly provided by Dr. Janet E. Foley, and from the Swedish dog by Dr. Anneli Bjöersdorff.

### 3.2. Ethics Statement

The experimental study in sheep was approved by the Norwegian Animal Research Authority.

### 3.3. 454 Genome Sequencing and Bioinformatics

Genomic DNA was sequenced on the Roche/454 Genome Sequencer as previously described [13,22], with genome coverage ranging from 31.3X to 72.1X. Briefly, regular read libraries were generated for *Ap*HZ, *Ap*Dog, *Ap*JM, *Ap*MRK, *Ap*-CRT35, *Ap*NorV1 and *Ap*NorV2. Additionally, 3 kb paired end libraries were made for *Ap*HZ and *Ap*MRK. Genome drafts were assembled using the CLC Genomics Workbench software suite (version 4.0–4.9) with default parameters: length fraction, 0.5; similarity, 0.8; and for paired end reads, minimum distance, 1,500/maximum distance, 4,500. Gapped drafts for the *Ap*MRK and *Ap*NorV2 genomes were generated by a combination of mapping and *de novo* assembly. Briefly, initial consensus sequences for each strain were obtained by mapping the respective reads against the fully annotated Sanger sequenced *Ap*HZ genome (GenBank CP000235). Regions with corresponding *de novo* contigs were manually identified and replaced with *de novo* contig sequences. Reads were again mapped to the resulting consensus sequences and underlying aligned reads were inspected for conflicts and gaps, which were manually corrected as described [13,22]. In this fashion, 9 finalized contigs were obtained for *Ap*MRK, and 23 contigs for *Ap*NorV2. Residual gaps correspond mainly to some *msp2/p44* gene clusters, the large R3/R4 repeat regions of the *virB6-4* gene [22], and large genome duplications/insertions. To perform the analysis presented in Figure 1, for each of these two strains, a contiguous consensus genome sequence was generated consisting of the finalized contigs joined by Ns and ordered according to the CP000235 reference genome. To obtain the loci depicted in Figure 3A for *Ap*CRT35 and *Ap*NorV1, consensus sequences were generated by mapping the respective reads against the corresponding regions in the *Ap*MRK and *Ap*NorV2 genome drafts, and conflicts and gaps were manually resolved as above.

Genome alignments (Figure 1) were conducted using MUGSY and displayed with GMAJ [24]. To align Roche/454 reads with assembled contigs (Figure 2) LASTZ running on a local instance of GALAXY [25,26,27] on the University of Florida high performance computer cluster was used (75% identity cutoff). The SAM-format output files were converted to BAM, sorted and indexed with SAMTOOLS [28] and displayed with ARTEMIS [29,30]. Amino acid sequences were aligned with MAFFT [31] and displayed with CHROMA [32]. The putative signal peptide region and TM segments highlighted in Figure 4 were predicted with SIGNALP and PSORT [33,34,35,36].

### 3.4. PCR Amplification

PCR products spanning *msp4* and the *drhm* gene (~680 bp and 390 bp, respectively; Fig 3B and 3C) were derived in a single reaction or separately using Takara’s PrimeSTAR GXL DNA Polymerase system (Clonetech Laboratories, Mountain View, CA, USA). Reactions contained 5 ng gDNA or 5–12 µL from very low concentration samples, and: 1.25 U polymerase, 1.0 mM MgCl_2_, 200 μM each dNTP, and 200 nM each primer, in total 50 μL. Universal primers for *msp4* were: AB1692, 5′-TAATGATGCGTCTGATGTTAGCG-3′, forward; AB1693, 5′-CACCACCTGCTATGTTTACACG-3′, reverse, and for *drhm*: AB1694, 5′-TATCTTAGCTCTCTCCACCATAG-3′, forward; AB1695, 5′-AACTAGACGATGATACACAAGATG-3′, reverse. Following the manufacturer’s recommendations, 3-step PCR was performed with 30 cycles of 10 s denaturing at 98 °C, 15 s annealing at 60 °C and 90 s extension at 68 °C. Final extension was for 5 min at 72 °C. PCR products were analyzed on 1.5% agarose gels alongside a 100 bp DNA ladder (New England Biolabs, Beverly, MA, USA). 

### 3.5. GenBank Accession Numbers

GenBank accession numbers for the *drhm* gene in *Ap*MRK, *Ap*CRT35, *Ap*NorV1 (*drhm*-a), *Ap*NorV1 (*drhm*-b) and *Ap*NorV2 are KF905325, KF905326, KF905327, KF905328 and KF905329, respectively.

## 4. Conclusions

These data showed significant differences between *A. phagocytophilum* genomes derived from different host animals and geographic locations. Comparative genomics revealed a consistent gene deletion in the strains infecting humans in the U.S. compared to many other global *A. phagocytophilum* strains. A PCR assay was developed to detect the presence of this gene, *drhm*. This could be applied rapidly for strain identification in epidemiological studies to track *A. phagocytophilum* strains with varying degrees of potential infectivity to humans.
